# The peculiar properties of pericentromeric regions of genomes and their influence on sex chromosome evolution

**DOI:** 10.1371/journal.pgen.1012333

**Published:** 2026-10-27

**Authors:** Deborah Charlesworth

**Affiliations:** Institute of Ecology and Evolution, School of Biological Sciences, University of Edinburgh, Edinburgh, United Kingdom; Indiana University Bloomington, UNITED STATES OF AMERICA

## Abstract

Results from recent genome sequences support the idea that the lack of recombination across extensive sex-linked regions (SLRs) in some species may be a pre-existing feature, due mainly to the sex-determining locus being located near a centromere. This would cause it to be affected by the “centromere-effect”, which ensures low recombination rates near centromeres. In such cases, it is unnecessary to invoke any active recombination suppression process, or to debate ideas about the types of selection involved. In this review, I outline the properties of genome regions that recombine rarely, including pericentromeric regions of chromosomes, and discuss SLRs that overlap with pericentromeric regions. Unsurprisingly, it is difficult to distinguish special features of such SLRs that would suggest they have undergone a recombination suppression process. The property most likely to be specific to completely SLRs is major gene loss from Y- or W-linked regions of genes found in their respective X- or Z-linked counterparts, and this asymmetry is observed in many species. However, if pericentromeric regions are characterised by ephemeral gene content and low gene expression, asymmetry between pericentromeric SLRs might be less pronounced than in SLRs in other genome regions, and similar Y and X degeneration levels are indeed documented in several species. Additionally, some such SLRs may not be completely sex-linked, which could explain findings of extensive SLRs with low Y-X (or W-Z) divergence.

## Introduction

It is widely accepted that “sex chromosomes originate from a pair of homologous autosomes and recombination between them is suppressed via inversions” [1]. However, this ignores the alternative possibility, originally proposed based on findings in *Aedes aegypti*, that a lack of recombination may simply be a pre-existing feature resulting from a sex-determining locus being located near a centromere [2,3]. As described below, genome sequence assemblies of species with separate sexes and genetic sex-determination suggest that this is a common situation, and it seems important to ask how it might affect the properties of sex-linked regions (SLRs) and our ability to discover and analyse them.

The “centromere effect”, which ensures low crossover rates (but does not necessarily suppress gene conversion events) in regions near centromeres, has long been known and reviewed in [4–6]. Extensive SLRs may therefore not always reflect recombination suppression that evolved in response to the presence of the sex-determining gene (or genes). There has been little discussion of how such a pericentromeric sex-linked region might evolve, compared with other genome regions that gain a sex-determining gene. The latter could evolve suppressed recombination and come to share properties with pericentromeric regions that recombine rarely. In order to understand the evolutionary causes of recombination suppression, properties that distinguish the two possibilities must be therefore be identified. Genome sequencing studies have only recently become able to study pericentromeric regions, largely because all types of nonrecombining regions accumulate repetitive sequences (see below), which makes it difficult to sequence and annotate them. These problems could be especially severe for regions in which a sex-determining factor is maintained polymorphic over a long evolutionary time (by a requirement that matings occur between males and females), allowing such an isolated Y- or W-linked region to accumulate many differences from their initially similar X- or Z-linked counterparts. Analyses of these genome regions should nevertheless advance our understanding of genome evolution and the evolution of SLRs. In order to describe these ideas briefly, I summarise the distinctive properties of pericentromeric regions of chromosomes in Table 1, and cite review papers, rather than explaining the predicted properties in detail; the table focuses on properties that are relevant for considering situations in which a species’ sex-linked region is associated with such a region.

**Table 1:** Comparison between the expected characteristics of pericentromeric regions and other genome regions with higher recombination rates, and their possible relevance for the evolution of sex-linked regions.

Characteristic	Pericentromeric region properties, relative to other genome regions	Effect on the evolution of sex-linked regions within pericentromeric regions, versus other genomic locations	References to predictions and examples
**Recombination (crossovers)**	Rare (but gene conversion can occur, exchanging short sequence regions). Lower GC-biased gene conversion rates, and thus low GC content.	Can maintain associations with the sex-determining locus across large physical distances. Sex-linkage becomes detectable based on sex differences in allele frequencies at linked polymorphic loci.	[24,36,47,84–86]
**Linkage disequilibrium (LD)**	Higher than in other genome regions, causing lower effective sizes and efficacy of selection.		
**TE density**	Higher, with potential for rapid density changes. Some repeat types have high GC contents, increasing GC contents.	Genetic degeneration may be faster due to deleterious effects of TEs on gene expression, including higher methylation, and heterochromatinization or condensation	[25,39,87–96]
**Gene density**	Lower, because TE and satellite densities are high, but many expressed genes may be present, but genes may be ephemeral	Genetic degeneration by mutations affecting gene functions may be slower. Gene presence/absence variation may create the appearance of degeneration of the X (or Z) haplotype, not just the Y (or W).	[30–32,97], degeneration of sex-linked genes is reviewed in [56]
**Gene expression**	Lower, due to effects of TEs on gene expression, including by creating very long introns, or because they are recent duplicates (short and with few exons)	Genetic degeneration may be faster due to weaker purifying selection as well as lower efficacy of selection.	[26,39,98–101]
**Rearrangements**	More likely, due to the presence of TEs and satellite repeats allowing ectopic exchanges, and effects are often less deleterious than in recombining regions. Regions near the centromeres may be “inversion hotspots”, between or within species, and pericentric inversions may occur within species.	Duplications are more likely, both into and from these regions. Gene losses may be less asymmetrical than in sex-linked regions in other genomic locations. Polymorphic rearrangements might pre-date sex-linkage.	[15,25,102]

## Direct and indirect evidence for low recombination rates in pericentromeric regions

The availability of single nucleotide polymorphisms (SNPs) from genome sequencing has recently provided DNA markers for detailed genetic mapping. Consistent with earlier results from model organisms including humans [7], and other species reviewed by [8,9], all or most chromosomes in many animals, plants and fungi have extensive regions with very low recombination rates. Marey maps [e.g., ref. [10] show genetic map positions that remain the same across physically large regions of the chromosome assemblies, and long-read sequencing that can identify the centromeres is revealing that these regions very often include the centromeres. Cytological approaches that can detect both centromeres and crossovers in suitable species detect similar patterns [11]. The sizes of such regions vary greatly between plant species [9], and even between closely related species such as *Drosophila yakuba* and *D. santomea* [12]. However, limited family sizes may not detect rare crossover events, and can thus underestimate recombination rates.

Indirect ways of recognising pericentromeric regions are therefore also important, and, for many organisms, they provide the only current evidence. Genome sequences are now making such data available, including phased data that can detect centromere positions [13,14], and different chromosome arrangements, e.g., [15], and assign variants to their different haplotypes [16], including in SLRs, e.g., [17–20], though satellite sequences can create biases in the representation of the two haplotypes [21]. As expected, linkage disequilibrium (LD) decays more slowly in regions that include centromeres than in other parts of the same chromosomes, for example in humans [22] and species in the plant genus *Spinacia* [23]. Recombination may not be completely lacking, as the associations between SNPs in genome sequences of individuals sampled from natural populations decay within these regions (and, as mentioned above, gene conversion can certainly sometimes occur). However, LD is affected by many factors, including recent demographic changes, and analyses of LD are unreliable in repetitive regions.

## Effects of low recombination rates on genome evolution, and the use of the predicted differences to indirectly recognise pericentromeric regions

Table 1 summarises several other effects of low recombination rates, which allow hitch-hiking processes (background selection and selective sweeps) that reduce effective population sizes (*N*_*e*_) across entire nonrecombining regions, weakening the efficacy of natural selection, including its ability to eliminate deleterious variants [24]. Repetitive sequences are predicted to accumulate (especially in noncoding regions) by genetic drift. Furthermore, TE insertions and expanded satellite arrays can cause ectopic and unequal exchanges involving similar sequences, creating deletions or other rearrangements that are deleterious when heterozygous; if crossovers are rare, selection against them is reduced [25]. Rather than repetitive sequences being the primary cause of low recombination rates, low rates are therefore permissive for their accumulation, and their accumulation rate can approach the insertion rate. These effects can largely explain the correlation between low recombination rates and repeat-richness, although repeat accumulation may further reduce recombination [26]. Repeat accumulation creates lower gene densities than elsewhere in the same chromosome (Table 1). However, unequal exchanges and processes such as rolling circle replication can also amplify sequences, which may then become fixed in a population by drift; array sizes in nonrecombining regions can become very large. Other processes include gene duplications into the regions from progenitors elsewhere in the genome. Gene densities therefore provide less reliable information about a region’s recombination rate than repetitive sequence densities, although these can also be difficult to interpret, as TE insertions can be followed by deletions and by nested insertions into them [27,28]. A lack of recombination is also associated with chromosomal rearrangements, because crossovers in heterozygotes can lead to duplications and deficiencies, see [29], and the absence of recombination removes this selection against them, allowing pericentric inversions to occur (see Table 1).

Overall, the processes listed in Table 1 predict generally lower expression levels of genes present in nonrecombining than in other genome regions, as they lead to extensive heterochromatic regions with high repressive methylation and histone modifications, often categorised as a distinct “B-type genomic compartment”. Nevertheless, these regions can include expressed genes [30–33], and higher methylation levels can sometimes be associated with high transcription levels, as reviewed in [34]. An important issue, however, is that not all annotated genes in current assemblies are complete and functional, especially in repeat-rich regions [21]. Their predicted low expression may cause preferential loss of function of duplicates in these regions (rather than the expected random retention of duplicated or progenitor copies [35]). Overall, genes in such regions may be ephemeral. It may therefore be difficult to distinguish between a low efficacy of selection due to a low effective population size (as reviewed in [36], and weak selective constraints due to low expression levels or over-representation of duplicated genes (Table 1), either of which will lead to rapid sequence evolution; this is distinct from the rapid evolution documented for the sequences likely to be involved in functional centromeres, including those of humans [37].

Pericentromeric regions can sometimes be recognised (and their extents determined) in genome sequences, especially long-read ones in which high repetitive sequence densities, low gene densities, and the presence of rearrangements are detectable [10]. However, pericentromeric regions probably rarely have clear boundaries, and different species differ in the characteristics that can provide the best clues. Recombination rates appear to change gradually with distance from centromeres [38], and properties such as gene densities might also change continuously; intermediate regions are indeed seen in several plants [39,40]. Moreover, subsequent changes in TE insertions may quickly obscure any sharp boundaries, as repetitive sequence densities are highly stochastic and can change rapidly. Finally, objective tests, such as change-point analyses of TE or gene densities, may be affected by several factors, including base composition differences (see Table 1).

Overall, densities of genes and repeats give the clearest signs of pericentromeric regions. Although the hitch-hiking processes mentioned in Table 1 cause low sequence diversity across entire nonrecombining regions, this signal cannot always be used. In some species, the pericentromeric regions have very low gene densities, so that hitch-hiking processes are ineffective [40,41], and in highly inbreeding species, recombinant genotypes are rare in all genome regions, due to high homozygosity [42,43], and therefore pericentromeric and other genome regions cannot be distinguished.

Some animal and plant species have holocentric chromosomes (defined as having multiple centromeric units on each chromosome, rather than a single centromere) and regional variation in recombination rates is documented, with crossovers much more often occurring in distal regions than elsewhere [44–46]. In the few species so far studied, crossovers are absent inside the centromeric units, but not in nearby genome regions. There are therefore no canonical centromere effects, but the chromosomal patterning of crossover probabilities can result in effects similar to those outlined above [47], though more studies of holocentric species are needed to assess this.

## Sex-linked pericentromeric regions

As many of the features outlined above are similar to those of extensive and old-established SLRs (reviewed in [48], a pericentromeric region that evolved a sex-determining factor, even recently, will show many of them. Properties such as repeat-richness and propensity to rearrange do not require complete absence of recombination, as illustrated by the “intermediate regions” mentioned above. Indeed, in maize and tomato recombination hotspots are detected in pericentromeric heterochromatin [49,50], consistent with the presence of recombination nodules in the tomato, although less frequently than in euchromatin [51]. Occasional recombinants were detected in the pericentromeric sex-determining region of *Populus tremuloides* [52], and the small spinach male-determining locus has higher repeat content than the rest of this chromosome arm, indicating some independence between the evolution of this region and the rest (which implies at least occasional recombination) [23,53].

Thus, recombination suppression in response to the appearance of a sex-determining factor may not be required. If repeat-richness is shared by both a species’ X- and Y-linked regions, with similar chromosomal compartmentalisation, the lack of recombination may be a pre-existing state, especially if all autosomes have extensive pericentromeric regions, making it plausible that sex-determining factors might sometimes evolve in such a region. Indeed, duplications into the regions have repeatedly created such factors (Table 2 includes several examples). The only completely Y- or W-linked region might sometimes be the sex-determining factor itself, and the rest of the region might simply recombine rarely. To understand the lack of recombination in parts of chromosomes carrying sex-determining loci, it is therefore necessary to consider whether any properties are specific to Y- or W-linked regions.

**Table 2:** Examples of species in which the sex-determining region is within a pericentromeric region, or is likely to be in such a region as it is physically near the centromere. Where available, the table includes estimated Y-X or W-Z (inter-gametolog) divergence (*K*_*S*_) in the oldest stratum; these are raw divergence values, not corrected for diversity within the individual sex chromosomes at the loci analysed, and they are therefore slight over-estimates.

Species	Heterogametic sex	Evidence for turnover?	Inter-gametolog raw divergence estimate (median or mean K_S_) in oldest stratum	References
**Plants**				
*Spinacia oleracea* (Chenopodiaceae)	Male	Yes (region is inserted into the Y chromosome)	~ 3.5%	[53,103,104]
*Rumex hastatulus* (Polygonaceae)	Male		~ 3%	[17,105]
*Carica papaya* (Caricaceae)	Male		s	[106,107]
*Populus* and *Salix* (Salicaceae)	Male or female	Yes (the Y- or W-linked sex-determining genes are partial duplications that inhibit autosomal genes involved in flower development)	~ 1-2%	[52,108–112]
*Itoa* (Salicaceae)	Male		4.2%	[113]
*Eucommia ulmoides* (Garryaceae)	Male	Yes		
*Hippophae species* (Elaeagnaceae)	Male		~1-2%	[114,115]
*Mercurialis annua* (*Euphorbiaceae*)	Male	Yes	1.1%	[116,117]
**Fish and amphibia**				
*Takifugu*	Male	Yes		[118]
*Hyporhamphus* species	Male or female	Yes	~ 3.5%	[119]
Zig-zag eel, *Mastacembelus armatus*	Male		—	[120]
Seahorse (*Hippocampus*)	Male	Yes	0.2% in *H. abdominalis*, 0.6 % in *H. erectus*	[121]
*Leptodactylus bufonius* (a frog)	Male		—	[122]
*Aedes aegypti* (a Dipteran insect)	Male	Likely (ancient duplication of a transformer-like gene, *Nix*, in the Y-linked region)	Unknown, but Fst is high across ~ 1/3 of the chromosome	[2,123,124]

## Do the pericentromeric regions of autosomes differ from the properties of such regions that overlap SLRs?

If a Y- or W-linked region evolves in a pericentromeric region whose repeat density is already near 100%, no increase after sex-linkage evolved will be detected, leaving it unclear whether recombination was subsequently suppressed completely (see the next section). However, in the plant *Rumex hastatulus*, many 1 Mb Y chromosome windows are entirely repetitive (unlike X chromosome windows) [17]. Moreover, even though full sex-linkage has led to low synonymous site divergence (averaging less than 3%), the Y and X gene orders are so different that the number of rearrangements involved cannot be determined. In a part that became sex-linked even more recently, due to an X-autosome fusion, the number of new rearrangements may not, however, exceed what might be expected based on rearrangements in the large autosomal pericentromeric regions (which may even include ectopic exchanges between the pericentromeric regions of different chromosomes, causing reciprocal translocations) [54]. Current evidence is scanty, but suggests that rearrangements may also be more abundant in Y-linked pericentromeric regions than those on the autosomes or the X, even though Y-linkage is not complete [55].

One major difference is expected, however: completely Y-linked regions undergo genetic degeneration, including eventual gene losses [56]. The reduced effective population size caused by selection in regions that recombine rarely was outlined above. In addition, populations with a 1:1 sex ratio include three times as many X chromosomes as Y chromosomes, further reducing the Y’s effective population size [57]. A lack of recombination with the X in males also keeps new mutations heterozygous, and therefore selection opposes genetic drift more weakly for Y-linked mutations than those in other genome regions, especially mutations that are largely recessive in their effects on fitness. As X-linked regions can recombine in females, generating homozygotes in which deleterious effects are expressed, they are expected to degenerate much less after sex-linkage evolves [58].

However, as already mentioned, coding sequences in pericentromeric regions might experience inherently weak selective constraints (for example, due to low expression levels). Mutant alleles might then drift to high frequencies, or even fixation. If genes in such regions are unusually prone to being lost, or to losing function, the asymmetry between Y- and X-linked regions might be smaller than in SLRs that are not pericentromeric, which might explain the roughly similar degeneration observed for both haplotypes of the SLRs of papaya [59] and the willow species in Table 2.

## Do sex-linked pericentromeric regions tend to expand and form younger evolutionary strata?

How might recombination be affected on a single chromosome that has evolved a sex-determining locus? Excluding situations involving a fusion with an autosome, which could affect recombination patterns, it is often suggested that nonrecombining sex-determining regions should expand over time, and that lack of such an expansion requires explanation in terms of evolutionary forces that have impeded it, maintaining a physically small region, as proposed to explain the lack of sex chromosome heteromorphism in the mosquito *Aedes aegypti* [60]. However, the occasional recombination in pericentromeric regions mentioned above may explain such situations. A more interesting question is how physically large SLRs that include the centromeres of the chromosomes might evolve. There are two possibilities: (i) a sex-determining factor in a nonpericentromeric region might trigger an expanded, completely sex-linked region overlapping the pericentromeric region, or (ii) a sex-determining factor in a pericentromeric region might lead to complete sex-linkage evolving across all or part of that region, and subsequently expand to include previously nonpericentromeric regions [61].

In either scenario above, rearrangements such as inversions may become fixed Y-X (or W-Z) differences that prevent crossovers, although predictions about rearrangements creating sex-linkage depend on what selective force, if any, favours closer linkage [62,63]. Analyses of differences between sex chromosome pairs, along with the arrangements of the homologous chromosomes of close outgroup species, allow inferences of whether Y- and W-linked regions rearrange more than X- or Z-linked ones, as appears to be the case.

Among rearrangements, inversions are most often discussed in relation to sex chromosomes [64], because they suppress recombination and can respond to potential selection pressures favouring lower recombination rates reviewed by [63]. In the human XY pair, there is support for the involvement of inversions in the formation of the most recent “strata”, adjoining the PAR [65]. However, recombination can be suppressed in specific genome regions by specific modifiers of recombination like those detected in yeast [66–68] and *D. melanogaster* [69], and other factors can affect crossover patterns [70], as noted by [71].

Recombination might, however, be inhibited directly near the boundary of a nonrecombining sex chromosome region. A pericentromeric region carrying a new sex-determining locus would show LD with sequence variants in the adjacent PAR, as sex-determining alleles are maintained by balancing selection, and such polymorphisms creates a peak of neutral polymorphism at closely linked sites [72,73]. The presence of such variation might inhibit recombination, extending the size of the nonrecombining region without any selection favouring suppressed recombination, although the effect of sequence variation on recombination rates is currently unclear [74]. The physical size of the region affected will be very small unless the recombination rate is extremely low [72,73]. Thus, this process might act within pericentromeric regions. There is little empirical evidence for this model, but species of interest would be ones whose completely SLRs were initially small parts of their pericentromeric regions, which later expanded across more of the region.

One species that could fit this scenario is *Silene latifolia*, whose XY pair includes a pericentromeric region with low gene densities in both the X and Y haplotypes, although the Y-linked region has lost many genes present on the X, and is clearly degenerated (the X and Y arrangements differ greatly, including by at least one pericentric inversion [20,75,76]. It has been proposed that recombination suppression was initially established within the pericentromeric region, and subsequently expanded [61]. However, the oldest stratum appears not to overlap that region, as (based on genes present on both the X and the Y) the X-linked genes with the highest synonymous site divergence from their Y alleles (*K*_S_ values) are in a region that still recombines in females. It remains unknown why and how the pericentromeric region of this species became fully sex-linked, though this presumably happened before the split of the five *Silene* species in section *Melandrium*, which share largely the same sex-linked region. Changes have not, however, stopped, as a region has recently become fully sex-linked in *S. latifolia* but is still pseudo-autosomal in its close relative, *S. dioica* [77,78].

The dioecious Cannabaceae species, *Cannabis sativa* and *Humulus* (hop), also have a repeat-rich fully sex-linked region (or old stratum) that includes the chromosome’s centromere, though with even higher divergence between Y and X alleles (*K*_S_ estimates are at least 20%; [55,79]. In these species, however, although the *K*_S_ values are too high for reliable estimates of the time when complete sex-linkage evolved (shortly before the two genera diverged), this apparently affected at least one entire arm of the chromosome, with no sign of a younger stratum at the *q* arm’s nonpericentromeric right-hand end. The PAR is on the *p* arm, which does include a younger stratum, with *K*_S_ values of only ~5%, and minor Y degeneration. The change from the adjoining old stratum occurs over a short physical distance, further arguing against a very slow process of expansion of the fully sex-linked region under the nonselective hypothesis.

Heteromorphism of sex chromosomes with extensive SLRs could reflect deletions after major gene losses from any type of SLR, not only large pericentromeric regions. The time course expected for a Y or W chromosome to undergo deletions and become visibly heteromorphic is currently still not completely clear, but such heteromorphism should often be associated with smaller Y or W sizes, compared with their X and Z counterparts, unless major repeat expansion occurs. Heteromorphism can potentially also arise after turnover events. If a new sex-determining factor evolves within a pericentromeric region, or duplicates into such a region, an extensive SLR could arise immediately. The X and Y haplotypes would initially be very similar, but they could differ if polymorphic chromosomal arrangements are present, as in some *Drosophila* sex chromosomes [80]. Such polymorphisms are found in many pericentromeric regions, for example in humans [22] and the plant species tomato [81] and spinach [23]. Furthermore, even without complete sex-linkage, the rarity of recombination would allow such chromosome variants to remain associated with one haplotype or the other for many generations, and heteromorphism could also quickly arise *de novo*, through major expansion of transposable elements or satellite repeats which (as noted above) can occur over short timescales in such regions, in either haplotype, though fixation is more likely in the Y or W population.

Pericentromeric regions are not the only situation in which a turnover event can create a physically large sex-linked region. The same can occur when crossovers are strongly localised in one sex. For instance, in a fish such as the guppy, whose chromosomes are acrocentric and crossovers in male meiosis are strongly localised to the noncentromere ends [82,83], a new male-determining factor is found in a distal region that recombines rarely. Owing to the very infrequent crossovers in pericentromeric regions, it is not currently known whether the centromere effect differs between the sexes.

## Conclusions and questions for future study

Some of the ideas outlined above are currently supported only weakly, and it is important to understand that they do not apply to all SLRs, and that there is a variety of sex chromosomes, as reviewed in ref. [1]. Recombination suppression has certainly sometimes evolved, including in Eutherian mammals, possibly involving establishment of Y-linked inversions [64]. An initial sex-linked locus outside the pericentromeric region of the sex chromosome could thus lead to recombination suppression by any of the mechanisms so far proposed, and this could extend the sex-linked region to include all or part of the pericentromeric region. The vast Y-linked region in the plant *Silene latifolia* appears to have evolved in this way [20]. Given enough evolutionary time, repetitive sequence accumulation and rearrangements in the pericentromeric portion would be expected to lead to the observed XY heteromorphism.

Several other areas where more studies are needed have already been mentioned, including the possibility that a larger proportion of genes in pericentromeric regions may be under less efficacious selective than genes in other genome regions. The purpose of reviewing these ideas is to suggest that they are plausible, and that, now that these genome regions can be studied, they should be developed and tested. Among further questions of interest are (i) whether gene duplications are commoner than elsewhere in genomes, forming “young genes” specific to single species (and whether some of these are pseudogenes), (ii) what proportions of genes have complete coding sequences and apparently intact 3´and 5´ sequences, and appear functional, versus fragmented or nonfunctional due to frame-shifts or stop codons, (iii) whether their expression level differ consistently, and (iv) whether rearrangements are present as polymorphisms in these regions. If pericentromeric regions prove to evolve differently from the rest of the genome, forming a distinct “genomic evolutionary compartment”, as well having special properties in the genomes of extant organisms, it will be important to know this.

As well as questions concerning pericentromeric regions in general, specific questions arise concerning sex-linked pericentromeric regions. Although it is now clear that such regions exist, they are usually described without considering the implication that large SLRs could potentially have evolved without selection due to interacting genes having favoured less recombination in the region. Related to this is the further interesting question of how often pericentromeric regions that occasionally recombine evolve complete loss of recombination after sex-linkage is established, and how this might happen.
